# Investigation of Molecular Alkali Tetrafluorido Aurates by Matrix‐Isolation Spectroscopy

**DOI:** 10.1002/chem.201904335

**Published:** 2019-10-24

**Authors:** Frenio A. Redeker, Mathias A. Ellwanger, Helmut Beckers, Sebastian Riedel

**Affiliations:** ^1^ Institut für Chemie und Biochemie Anorganische Chemie Freie Universität Berlin Fabeckstr. 34/36 14195 Berlin Germany

**Keywords:** fluorine chemistry, gold chemistry, laser-ablation, matrix-isolation spectroscopy, quantum-chemical calculations

## Abstract

Molecular alkali tetrafluorido aurate ion pairs M[AuF_4_] (M=K, Rb, Cs) are produced by co‐deposition of IR laser‐ablated AuF_3_ and MF in solid neon under cryogenic conditions. This method also yields molecular AuF_3_ and its dimer Au_2_F_6_. The products are characterized by their Au–F stretching bands and high‐level quantum‐chemical calculations at the CCSD(T)/triple‐ζ level of theory. Structural changes in AuF_4_^−^ associated with the coordination of the anion to different alkali cations are proven spectroscopically and discussed.

## Graphical abstract

**Going for gold**! M[AuF_4_] (M=K, Rb, Cs) are produced by co‐deposition of IR laser‐ablated AuF_3_ and MF in solid neon under cryogenic conditions. This method also yields molecular AuF_3_ and its dimer Au_2_F_6_. Structural changes in AuF_4_^−^ associated with the coordination of the anion to different alkali cations are proven spectroscopically and discussed (see figure).

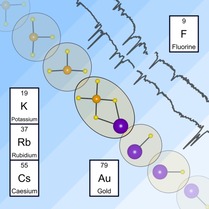

Isolation of molecular ions and ion pairs has always been a challenge in matrix‐isolation spectroscopy. One method that is capable of producing mostly radicals but also small amounts of anions and cations is by passing gas‐mixtures through a microwave discharge.^1^ Another way to produce ions is to use laser‐ablation of metal targets which produces electrons and thereby anionic species like the free trifluoride ion (F_3_^−^).^2^ A long known method to isolate molecular ion pairs is by evaporation of a salt at high temperatures in a Knudsen cell.^3^ However, this approach is elaborate and needs long deposition times. Recently, free ions in addition to ion pairs were isolated in rare‐gas matrices by pulsed IR‐laser deposition of salt targets.^4^, ^5^ This method produces significantly higher yields in a fraction of the time needed for thermal evaporation and is much simpler to control.

Herein, we report a simple method that allows for the reaction of two crystalline nonvolatile reactants by laser‐ablation of a mixed salt target material. With that method it was possible to produce and characterize molecular alkali tetrafluorido aurates (M[AuF_4_], M=K, Rb, Cs) for the first time by reaction of laser‐ablated alkali fluorides (MF) with gold trifluoride (AuF_3_) under cryogenic conditions. The Au–F stretches of such molecules are slightly dependent on the alkali metal and are shown to be in excellent agreement with high level quantum‐chemical calculations. To the best of our knowledge, Raman and IR studies have so far only been published on the crystalline bulk material of M[AuF_4_].^6^, ^7^

In IR spectra recorded after co‐deposition of laser‐ablated MF/AuF_3_ (M=K, Rb, Cs) with excess neon at 6 K several bands were observed in the Au–F stretching region (Figure 1). The position of some of those bands were found to be alkali metal‐dependent and some are metal‐independent. Two metal‐independent bands at 694 and 692 cm^−1^ are, according to Wang et al., assigned to AuF_3_.^8^, ^9^ Two further weak bands at 655 and 494 cm^−1^ in this previous work were attributed to Au_2_F_6_ obtained by evaporation of solid AuF_3_ in a Knudsen cell.^8^, ^9^ In our spectra, the 655 cm^−1^ band is not present. Instead we observed four strong bands at 665, 660, 494, and 492 cm^−1^ associated with the four strongest stretching bands of Au_2_F_6_ (*D*_2*h*_): the in‐phase antisymmetric (b_2u_) and the out‐of‐phase symmetric (b_3u_) stretching modes of the terminal F atoms, and the in‐phase antisymmetric (b_3u_) and the out‐of‐phase symmetric (b_2u_) stretching modes of the bridging F atoms in descending order. A comparison of the calculated Au_2_F_6_ vibrational spectrum with our experimental results is shown in Table S1 in the Supporting Information. Both sets of bands, due to AuF_3_ and Au_2_F_6_ diminish under UV light (*λ*=273 nm), whereby the AuF_3_ bands are more sensitive to irradiation.

**Figure 1:**
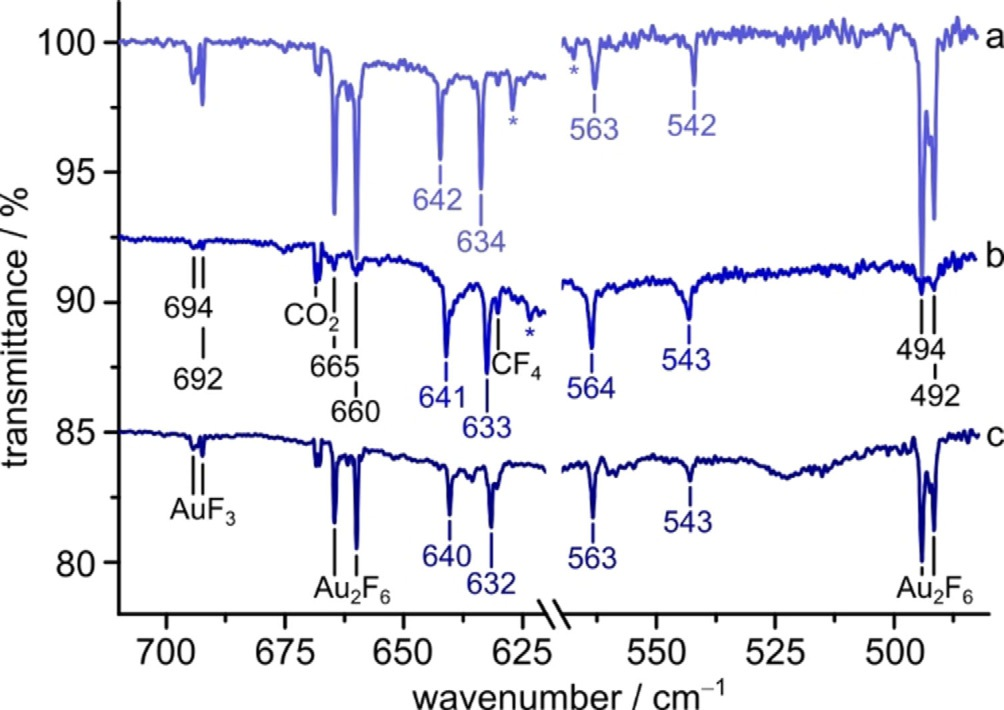
Au–F stretching region of the Ne‐matrix IR spectra obtained by laser‐ablation of solid mixtures of MF (M=K, Rb, Cs) and AuF_3_ (2–3 %) after irradiation with UV light (*λ*=273 nm, 5 min). a) KF, b) RbF, and c) CsF. The region of oligo‐ and polymeric M[AuF_4_] is not shown for clarity. Bands due to impurities that are also found in the IR spectra of pure laser‐ablated MF ion pairs in solid neon are marked by an asterisk.

Prior to irradiation a band at 562 cm^−1^ was present in all spectra, even after deposition of pure alkali metal fluorides MF and is known to be associated with the antisymmetric F_3_^−^‐stretch in MF_3_.^4^ The presence of MF_3_ ion pairs indicates the sequence of reactions (1)–(3) during laser‐ablation and matrix‐isolation of alkali fluorides. The MF_3_ band disappears completely by irradiation with UV light (*λ*=273 nm, 5 min). Its behavior is therefore very different from the irradiation resistant bands at 563–564 cm^−1^ (cf. Figures S1 and S2, Supporting Information).

(1) $$\mathrm{MF}\overset{hv}{\underset{}{\to }}M+F$$

(2) $$F+F\to F{}_{2}$$

(3) $$\mathrm{MF}+F{}_{2}\to \mathrm{MF}{}_{3}$$

These latter bands show a slight shift depending on the alkali metal M and they are part of a set of four bands that did not appear in the spectra obtained with pure alkali metal fluorides (MF). These findings suggest that the carrier of the four bands is a reaction product of the reactants MF and AuF_3_, most likely molecular alkali tetrafluorido aurate.

Calculations at the CCSD(T) level of theory suggest that molecular M[AuF_4_] has a *C*_2*v*_ minimum structure of a distorted square‐planar tetrafluorido aurate with two bridging fluorido ligands to the alkali metal (Figure 2). Compared with the free tetrafluorido aurate anion, the angle between the terminal fluorine atoms in the ion pairs almost remains 90°, while the angle between the *μ* ^2^‐F atoms is decreased (87°). The terminal Au−F bonds in the ion pairs are shortened whereas the Au−F′ bonds to the bridging fluorine atoms are elongated. This trend is also reflected in the calculated harmonic frequencies of the ion pairs M[AuF_4_] (Table 1): The terminal Au–F stretches (a_1_, b_1_) are predicted to appear 45–50 cm^−1^ higher and the bridging Au–F′ stretches (a_1_, b_1_) 40–60 cm^−1^ lower than the AuF_4_^−^ (e_u_) stretch. In the series M[AuF_4_] (M=K, Rb, Cs) the terminal Au–F bonds slightly decrease from Cs to K, whereas the Au–F′ bonds get longer, following the increase of the Lewis acidity of the alkali metal cations (Cs^+^<Rb^+^<K^+^). The observed blueshift of the terminal Au–F stretches at 640–642 and 632–634 cm^−1^ and the redshift of the Au–F′ bands at 563–564, and 542–543 cm^−1^ in the series from Cs to K is excellently matched by the calculated spectra (Figure 1, Table 1). The IR spectra of crystalline Cat[AuF_4_] (Cat^+^=Cs^+^, Me_4_N^+^, Et_4_N^+^)^7^ show only one AuF_4_^−^ band at 598 cm^−1^ indicating that in the crystal the *D*_4*h*_ symmetry of the anion is preserved.

**Figure 2:**
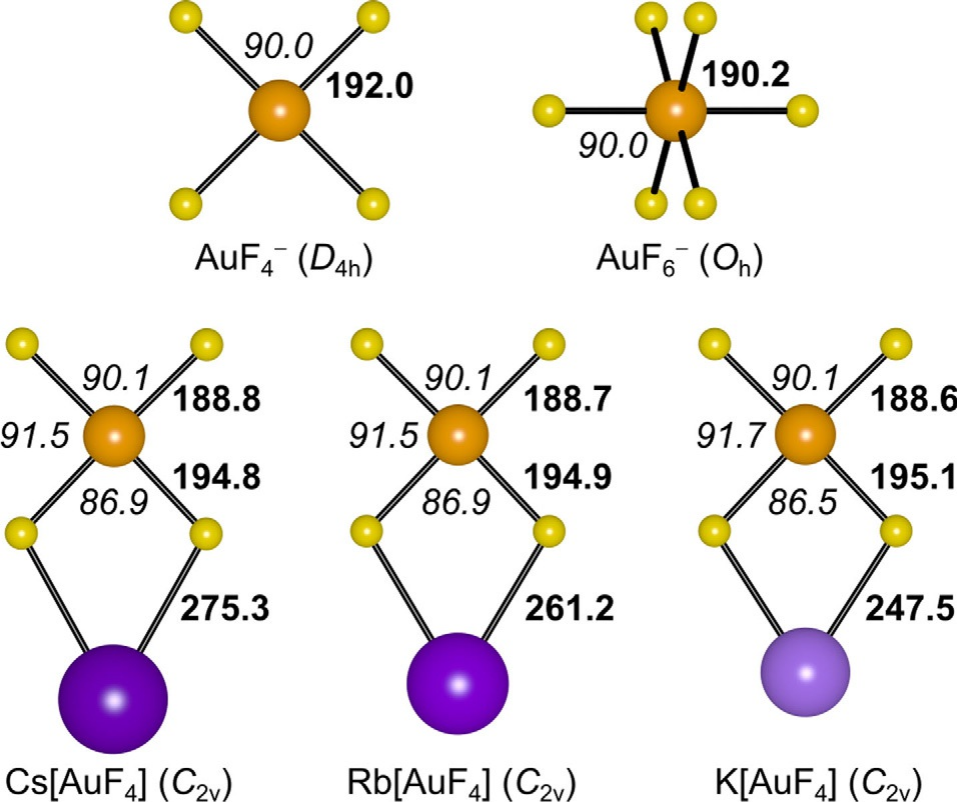
Structures of the M[AuF_4_] ion pairs (M=K, Rb, Cs) and the free anions AuF_4_^−^ and AuF_6_^−^ obtained at the CCSD(T)/def2‐TZVPP (M[AuF_4_]) and CCSD(T)/def2‐TZVPPD (free anions) levels of theory. Bold and italic numbers indicate bond lengths [pm] and bond angles [°], respectively.

**Table 1:** Comparison of selected experimental IR bands in neon with calculated harmonic frequencies [cm^−1^] of M[AuF_4_] ion pairs (M=K, Rb, Cs) and the free ions AuF_4_^−^ and AuF_6_^−^.

Species	Sym	B3LYP		SCS‐MP2		CCSD(T)		Exptl.
K[AuF_4_]^[a]^	b_1_	510	(49)	556	(69)	554	(57)	542
	a_1_	531	(107)	568	(133)	571	(121)	563
	b_1_	617	(97)	661	(101)	655	(99)	634
	a_1_	625	(90)	666	(92)	661	(89)	642
Rb[AuF_4_]^[a]^	b_1_	512	(46)	560	(65)	558	(53)	543
	a_1_	533	(106)	572	(133)	574	(119)	564
	b_1_	615	(98)	660	(102)	654	(100)	633
	a_1_	623	(95)	664	(98)	660	(96)	641
Cs[AuF_4_]^[a]^	b_1_	511	(41)	560	(58)	557	(47)	543
	a_1_	534	(115)	572	(142)	575	(128)	563
	b_1_	615	(94)	659	(99)	653	(97)	632
	a_1_	622	(101)	663	(104)	659	(102)	640
AuF_4_^−[b]^	e_u_	586	(350)	622	(401)	615		n.o.
AuF_6_^−[b]^	t_1u_	621	(439)	647	(418)	651		n.o.

[a] def2‐TZVPP basis set. [b] def2‐TZVPPD basis set. Calculated intensities in parentheses (km mol^−1^). Species that were not observed experimentally are indicated with n.o. (not observed).

The assignment of the stretching bands of the M[AuF_4_] ion pairs was further confirmed by IR spectra obtained after pulsed laser deposition of KF/K[AuF_4_] mixtures (Figure S1, Supporting Information), and CsF/Cs[AuF_6_] mixtures (Figure S2) in excess neon. The solid M[AuF_6_] salt was found to eliminate fluorine (F_2_) during laser‐ablation to yield matrix‐isolated M[AuF_4_] ion pairs [Eq. (4)]. With Cs[AuF_6_], for example, Cs[AuF_4_] and particularly high amounts of CsF_3_ ion pairs were obtained (Figure S2, Supporting Information). At the SCS‐MP2 level, reaction (4) is endergonic by Δ*G* (0 K)=154 to 157 kJ mol^−1^ for M=K, Rb, Cs (Table S(4).

(4) $$M\left[\mathrm{AuF}{}_{6}\right]\overset{hv,\;\Delta T}{\underset{}{\to }}M\left[\mathrm{AuF}{}_{4}\right]+F{}_{2}$$

The free anions AuF_4_^−^, predicted at 615 cm^−1^, and AuF_6_^−^, predicted at 651 cm^−1^ (CCSD(T)/def2‐TZVPPD, Table 1), were not observed in any of the experiments. It is, however, possible that the e_u_ stretch of the free AuF_4_^−^ ion overlaps with a broad band of oligomeric (M[AuF_4_])_*n*_ as shown in Figure S1, Supporting Information. Matrix‐isolation of laser‐ablated CsF/Cs[AuF_6_] did neither yield free AuF_6_^−^ nor the molecular ion pair Cs[AuF_6_].

In the present study, we report the complete sets of experimental IR stretching bands of molecular Au_2_F_6_ and M[AuF_4_] ion pairs with M=K, Rb, and Cs for the first time. These species were produced by laser‐ablation of solid mixtures of MF salts with AuF_3_ and isolated in solid neon under cryogenic conditions. The metal dependence of the IR active Au–F stretches in the ion pairs for the different alkali metals is fully consistent with their structural changes obtained by high‐level quantum‐chemical calculations. With these results at hand, we have shown that pulsed‐laser deposition from a mixed salt target is an excellent method to facilitate the reaction of two crystalline nonvolatile reactants under cryogenic conditions. By this new approach, using mixed salt targets, the investigation of larger ion paired species becomes viable.

## Experimental Section

Matrix‐isolation experiments were performed using a self‐built matrix chamber in which a vacuum of at least 6×10^−6^ mbar was maintained by an oil diffusion pump connected to a rotary vane pump. The matrix support was kept at a temperature of 6 K using a *Sumitomo Heavy Industries* cold head with a helium compressor unit. IR spectra were recorded using 1000 scans at a resolution of ≤0.5 cm^−1^ on a *Bruker Vertex 80v* FTIR vacuum spectrometer equipped with a KBr beam splitter and a liquid nitrogen cooled MCT detector (4000‐350 cm^−1^). In a typical experiment 97–98 % MF (M=K, Rb, Cs) and ≈2–3 % of a fluorido gold species (AuF_3_, M[AuF_4_], or M[AuF_6_]) were mixed and ground under an argon atmosphere and subsequently pressed into a cylindric pellet using a hydraulic lab press. The target was mounted onto a rotatable target holder and transferred into the matrix chamber. The solid MF/AuF_3_ mixture was evaporated using a focused pulsed Nd:YAG IR laser (1064 nm) with pulse energies of ≤50 mJ and a pulse length of 3–7 ns, and co‐deposited with pure neon at 6 K using deposition times varying between 90 and 180 min.

AuF_3_ and M[AuF_4_] were prepared as published.^7^ For the Cs[AuF_6_] preparation, Cs[AuF_4_] (200 mg, 0.49 mmol) was dissolved in anhydrous HF. Fluorine (2 bar, 25 equiv) was added and the mixture was irradiated with UV light for 12 h under constant stirring. Finally, excess F_2_ and anhydrous HF were removed at low pressure to obtain the product Cs[AuF_6_] in quantitative yields.

Calculations were carried out at the B3LYP,^10^ SCS‐MP2,^11^ and CCSD(T) levels (M[AuF_4_], M=K, Rb, Cs) using the Orca 4.0.1^12^ program package. CCSD(T) calculations for the free anions [AuF_4_]^−^ and [AuF_6_]^−^ were performed using Molpro 2015.1.^13^ The frozen core approximation was applied in all SCS‐MP2 and CCSD(T) calculations. The ion pairs M[AuF_4_] were calculated using def2‐TZVPP^14^ basis sets of triple‐ζ quality for all atoms. These basis sets include effective core potentials for Rb (ECP‐28),^15^ Cs (ECP‐46),^15^ and Au (ECP‐60).^16^ For the free anions AuF_4_^−^ and AuF_6_^−^, def2‐TZVPPD^17^ basis sets with additional diffuse functions were used for Au and F.

## Conflict of interest

The authors declare no conflict of interest.

## Supporting information

As a service to our authors and readers, this journal provides supporting information supplied by the authors. Such materials are peer reviewed and may be re‐organized for online delivery, but are not copy‐edited or typeset. Technical support issues arising from supporting information (other than missing files) should be addressed to the authors.

Supplementary
