# Supplementary material for: Investigation of Molecular Alkali Tetrafluorido Aurates by Matrix‐Isolation Spectroscopy
Source: Chemistry. 2019 Oct 24;25(66):15059–61. doi: 10.1002/chem.201904335 (PMC6900187; doi:10.1002/chem.201904335)
Supplement: Supplementary file 1 — Supplementary [file CHEM-25-15059-s001.pdf]

CHEMISTRY
A European Journal

Supporting Information

Investigation of Molecular Alkali Tetrafluorido Aurates by Matrix-Isolation Spectroscopy

Frenio A. Redeker, Mathias A. Ellwanger, Helmut Beckers, and Sebastian Riedel*[a]

chem_201904335_sm_miscellaneous_information.pdf

S1. Additional Figures and Tables 1
S2. Calculated CCSD(T) Structures and Vibrational Frequencies 3

S1. Additional Figures and Tables

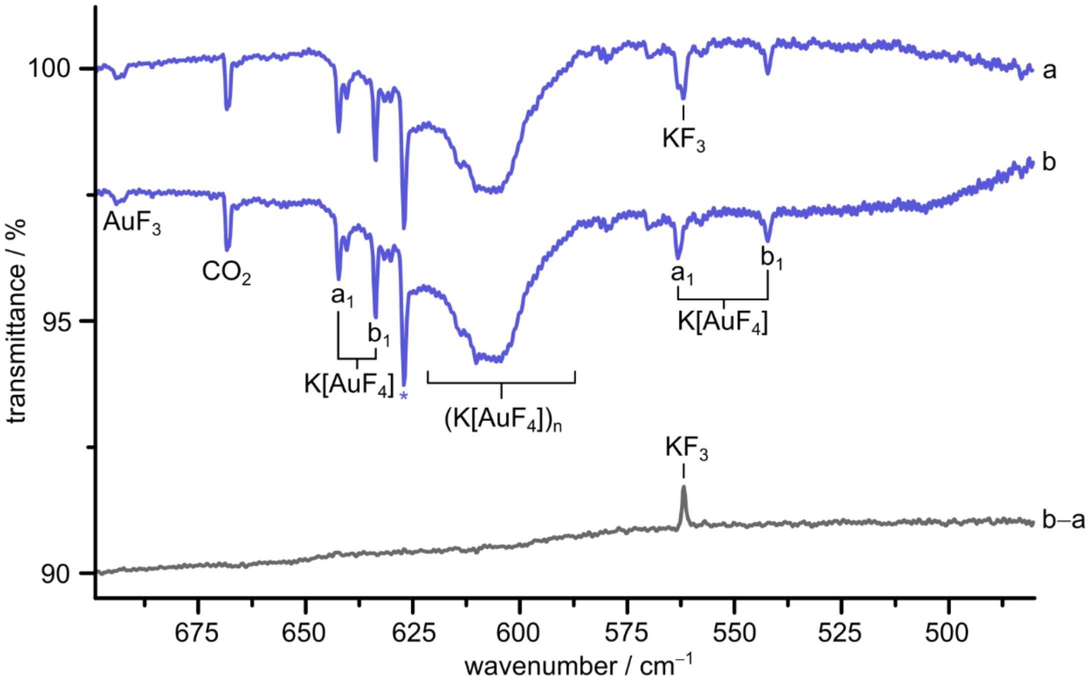

Figure S1. IR spectra in the Au−F stretching region obtained after co-deposition of laser-ablated KF with K[AuF$_4$] (3 %) in solid neon at 6 K, a) after deposition b) after irradiation with UV light (λ = 273 nm, 35 min), b−a) difference spectrum of b divided by a. Bands due to impurities that are also found in the IR spectra of pure laser-ablated KF in neon are marked with an asterisk.

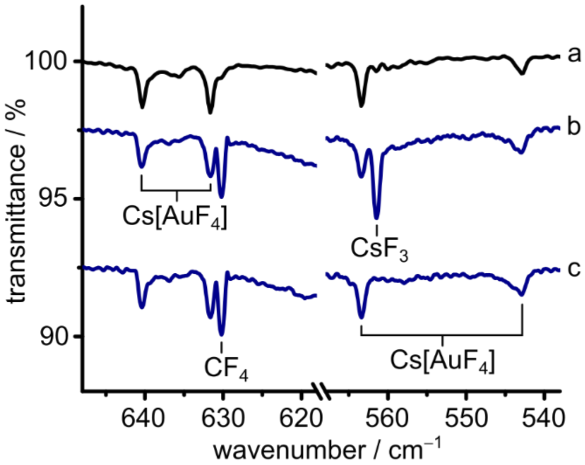

Figure S2. IR spectra obtained after a) co-deposition of laser-ablated CsF with AuF$_3$ (2.5 %) in excess neon at 6 K, b) co-deposition of CsF with CsAuF$_6$ (2 %) in excess neon at 6 K, and c) after irradiation of b (λ = 273 nm, 10 min).

Table S1. Comparison of calculated stretching frequencies of Au_2F_6 by Wang *et al.* with experimental band positions from the present study (cm^{-1}).

Sym.	Exp. in neon[a]	SCS-MP2/def2-QZVPP[b,c]	
b_{1g}	n.o.	459	(0)
a_g	n.o.	505	(0)
b_{2u}	492	507	(25)
b_{3u}	494	508	(324)
b_{1g}	n.o.	678	(0)
b_{3u}	660	683	(104)
b_{2u}	665	687	(96)
a_g	n.o.	689	(0)

[a] This work. [b] X. Wang, L. Andrews, K. Willmann, F. Brosi, S. Riedel, *Angew. Chem. Int. Ed.* **2012**, *51*, 10628. [c] Numbers in parentheses are calculated intensities (km mol^{-1}).

Table S2. Zero-point energy (ZPE) corrected free enthalpy ΔG (0 K) for selected reactions computed at the SCS-MP2 level of theory (kJ mol^{-1}).

$K[AuF_4]$	$\longrightarrow$	AuF_3	+	KF	364[a]
$Rb[AuF_4]$	$\longrightarrow$	AuF_3	+	RbF	370[a]
$Cs[AuF_4]$	$\longrightarrow$	AuF_3	+	CsF	355[a]
$K[AuF_6]$	$\longrightarrow$	$K[AuF_4]$	+	F_2	154[a]
$Rb[AuF_6]$	$\longrightarrow$	$Rb[AuF_4]$	+	F_2	157[a]
$Cs[AuF_6]$	$\longrightarrow$	$Cs[AuF_4]$	+	F_2	156[a]
AuF_6^-	$\longrightarrow$	AuF_4^-	+	F_2	172[b]
$K[AuF_6]$	$\longrightarrow$	AuF_5	+	KF	432[a]
$Rb[AuF_6]$	$\longrightarrow$	AuF_5	+	RbF	440[a]
$Cs[AuF_6]$	$\longrightarrow$	AuF_5	+	CsF	425[a]

[a] def2-TZVPP basis set. [b] def2-TZVPPD basis set.

S3. Calculated CCSD(T) Structures and Vibrational Frequencies

AuF_4^- (D_{4h})*, $^1A_{1g}$, CCSD(T)/def2-TZVPPD

Au	0.000000	0.000000	0.000000
F	0.000000	1.920412	0.000000
F	1.920412	0.000000	0.000000
F	0.000000	-1.920412	0.000000
F	-1.920412	0.000000	0.000000

b_{1u}	183
b_{1g}	220
a_{2u}	233
e_u	253
e_u	253
b_{2g}	573
a_{1g}	593
e_u	615
e_u	615

AuF_6^- (O_h)*, $^1A_{1g}$, CCSD(T)/def2-TZVPPD

Au	0.000000	0.000000	0.000000
F	0.000000	0.000000	-1.901575
F	0.000000	0.000000	1.901575
F	1.901575	0.000000	0.000000
F	0.000000	1.901575	0.000000
F	-1.901575	0.000000	0.000000
F	0.000000	-1.901575	0.000000

t_{2g}	212
t_{2g}	212
t_{2g}	212
t_{2u}	237
t_{2u}	237
t_{2u}	237
t_{1u}	277
t_{1u}	277
t_{1u}	277
a_{1g}	596
e_g	600
e_g	600
t_{1u}	651
t_{1u}	651
t_{1u}	651

$K[AuF_4]$ (C_{2v})*, $^1A_1$, CCSD(T)/def2-TZVPP

Au	0.000000	0.000000	0.000000
F	0.000000	0.000000	1.951106
F	1.885460	0.000000	-0.055860
F	-0.060435	0.000169	-1.885350
F	-1.947428	-0.000034	0.120236
K	-2.400660	0.000704	2.553334

b_2	46	(6)
b_1	147	(4)

a_1	153	(17)
a_2	183	(0)
b_2	232	(21)
a_1	244	(1)
b_1	291	(5)
a_1	328	(34)
b_1	554	(57)
a_1	571	(121)
b_1	655	(99)
a_1	661	(89)

Rb[AuF$_4$] (C_{2v})*, $^1A_1$, CCSD(T)/def2-TZVPP

Au	0.000000	0.000000	0.000000
F	0.000000	0.000000	1.948680
F	1.886827	0.000000	-0.048321
F	-0.053851	-0.000009	-1.886595
F	-1.945796	-0.000034	0.105483
Rb	-2.514992	0.000000	2.655091

b_2	38	(2)
a_1	115	(12)
b_1	128	(2)
a_2	183	(0)
b_2	231	(20)
a_1	242	(0)
b_1	283	(4)
a_1	309	(25)
b_1	558	(53)
a_1	574	(119)
b_1	654	(100)
a_1	660	(96)

Cs[AuF$_4$] (C_{2v})*, $^1A_1$, CCSD(T)/def2-TZVPP

Au	0.000000	0.000000	0.000000
F	0.000000	0.000000	1.947907
F	1.887461	0.000000	-0.048403
F	-0.054233	-0.000013	-1.887269
F	-1.945032	0.000000	0.105816
Cs	-2.626548	-0.000367	2.773286

b_2	35	(1)
a_1	96	(12)
b_1	111	(1)
a_2	183	(0)
b_2	231	(18)
a_1	239	(1)
b_1	272	(4)
a_1	293	(30)
b_1	557	(47)
a_1	575	(128)
b_1	653	(97)
a_1	659	(102)

* Calculated structures and harmonic frequencies are displayed in Å and cm^{-1}, respectively.
